# Recent advances in understanding oxidative stress in sepsis: pathogenic roles and antioxidant therapeutic prospects - a narrative review

**DOI:** 10.3389/fphar.2025.1695992

**Published:** 2025-11-10

**Authors:** Yijuan Lin, Ahmad Alhaskawi, Lin Chen, Safwat Adel Abdo Moqbel

**Affiliations:** 1 Department of Emergency, Hangzhou Linping District First People’s Hospital, Hangzhou, China; 2 Department of Orthopedics, the First Affiliated Hospital of Zhejiang University School of Medicine, Hangzhou, China; 3 Department of Emergency, the Second Affiliated Hospital of Zhejiang University School of Medicine, Hangzhou, China

**Keywords:** sepsis, oxidative stress, superoxide dismutase, vitamins, glutathione

## Abstract

Sepsis remains a major global health challenge, exerting a particularly severe toll in low- and middle-income countries. Despite advances in antimicrobial and supportive care, sepsis continues to defy effective control due to its complex pathophysiology and multi-organ involvement. Central to this complexity is a dysregulated host response, driven by hyperinflammation, immune suppression, and profound mitochondrial and metabolic dysfunction. A critical mediator of this dysregulation is oxidative stress, which exacerbates cellular injury through reactive oxygen and nitrogen species, disrupting mitochondrial integrity and redox balance. This review synthesizes current insights into the mechanistic interplay between oxidative stress, mitochondrial dysfunction, and immunopathology in sepsis. It further evaluates the therapeutic potential of endogenous antioxidants, such as superoxide dismutase, catalase, and glutathione, as well as exogenous agents including vitamins A, C, E, selenium, omega-3 fatty acids, melatonin, and carnosine. While translational gaps persist, particularly in dosing, timing, and patient stratification, emerging strategies including mitochondria-targeted antioxidants, nanotherapeutics, and biomarker-guided interventions hold promise for restoring redox homeostasis and improving clinical outcomes. This review aims to serve as a contemporary resource for researchers and clinicians striving to decode the oxidative basis of sepsis and accelerate the development of precision antioxidant therapies.

## Introduction

**FIGURE 1:**
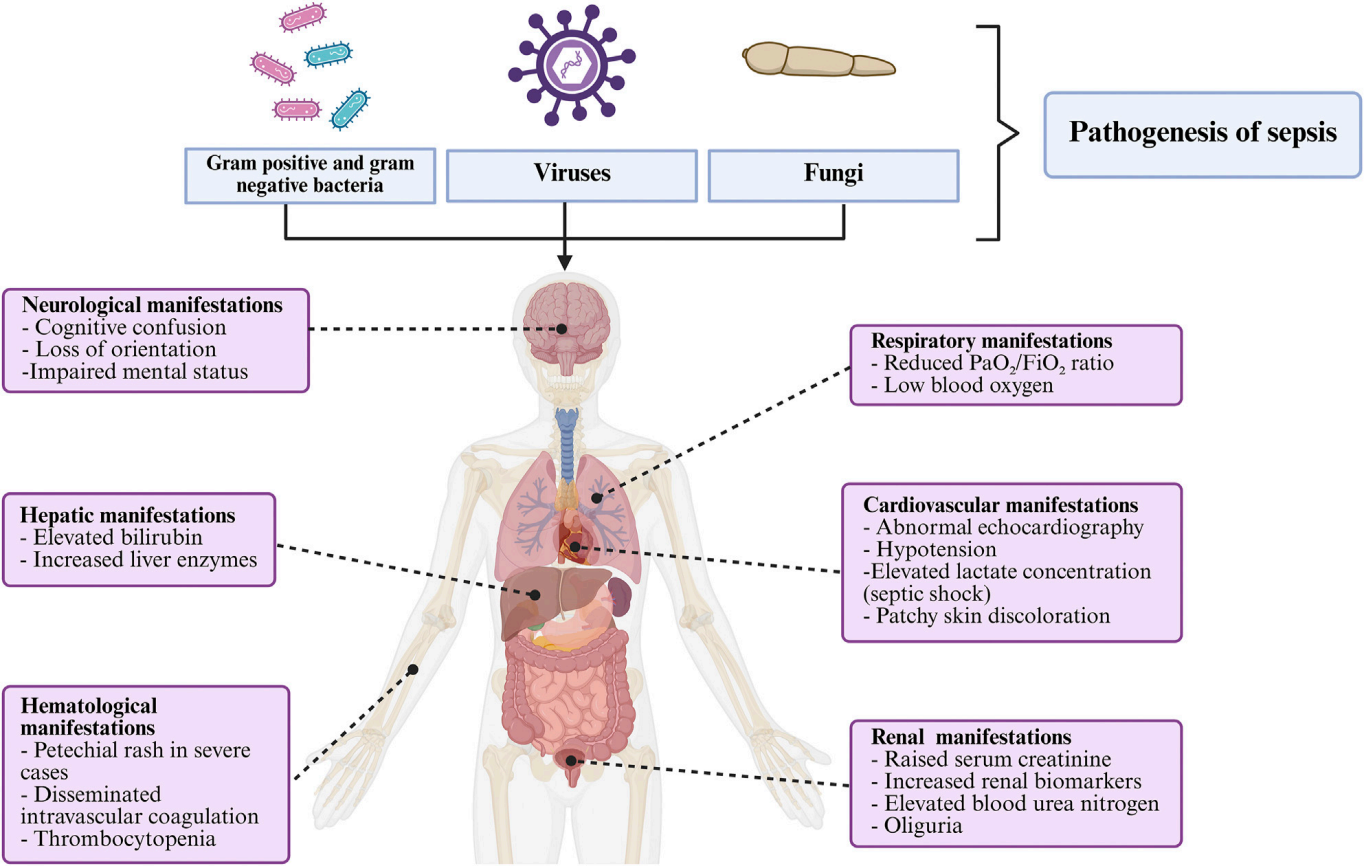
The pathological and clinical features of sepsis.

**FIGURE 2:**
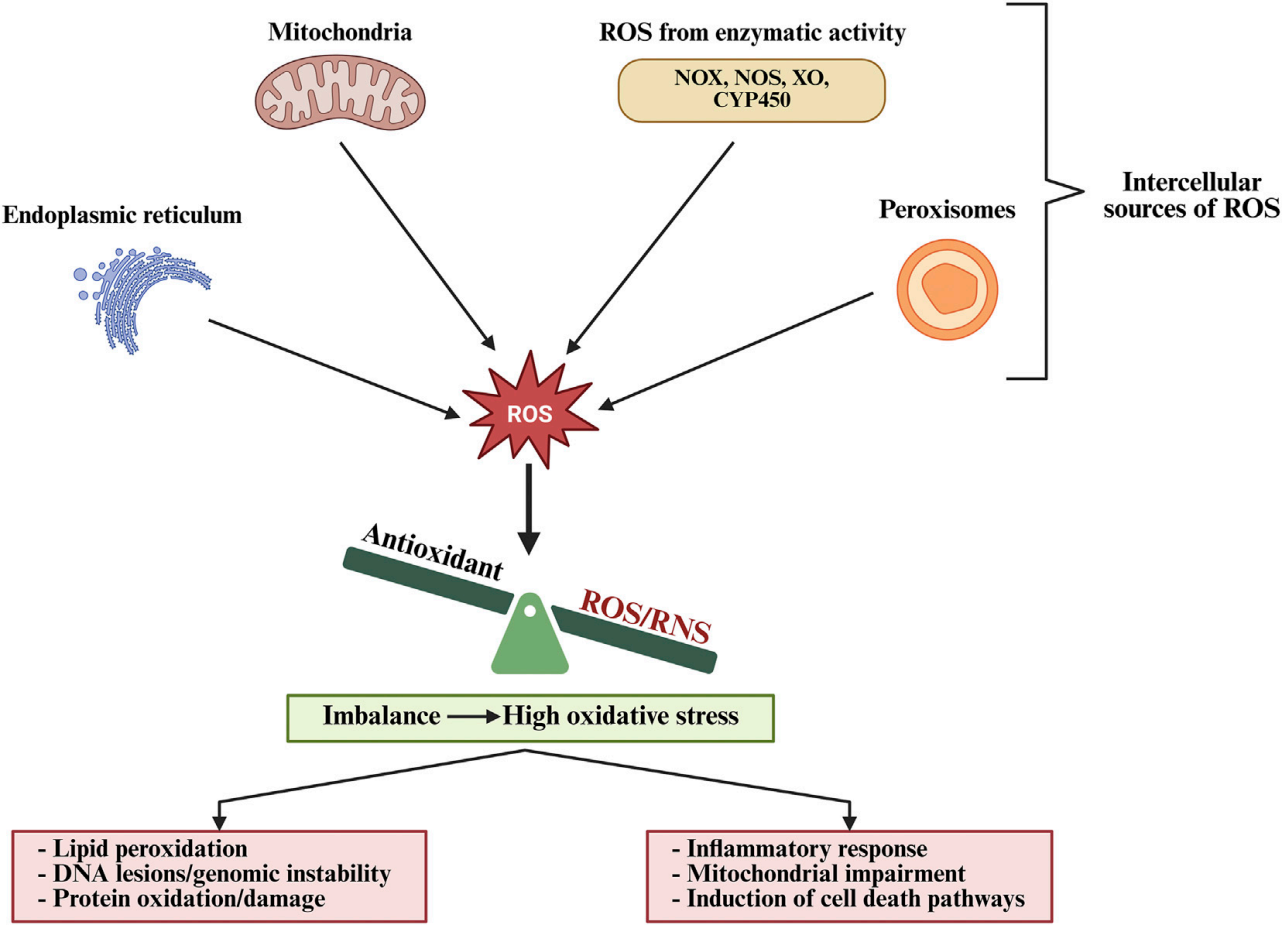
Intracellular sources and consequences of reactive oxygen species (ROS) generation.

Sepsis is a life-threatening organ dysfunction triggered by a dysregulated host response to infection, and remains a critical challenge in intensive care medicine. Despite advances in antimicrobial therapy and supportive care, sepsis continues to result in high morbidity and mortality, with an estimated 48.9 million cases and 11 million deaths worldwide in 2017, accounting for nearly 20% of all global mortality, disproportionately affecting low- and middle-income countries (Rudd et al., 2020). Sepsis is primarily triggered by a wide range of microorganisms, including Gram-positive and Gram-negative bacteria, viruses, fungi, and, less commonly, parasites, with bacterial infections accounting for the majority of cases globally (Neri et al., 2015). Clinically, sepsis manifests with nonspecific signs, including fever or hypothermia, tachycardia, tachypnea, and leukocytosis or leukopenia. As the disease progresses, it leads to hypotension, altered mental status, coagulopathy, and hyperlactatemia, reflecting systemic inflammation and tissue hypoperfusion. One of the hallmark challenges in managing sepsis is its ability to affect multiple organ systems simultaneously (Figure 1). Common complications include acute kidney injury, acute respiratory distress syndrome (ARDS), hepatic dysfunction, myocardial depression, and coagulopathy, often resulting in disseminated intravascular coagulation (DIC). The central nervous system may also be compromised, leading to sepsis-associated encephalopathy, which can persist long after infection resolution (Peerapor et al., 2019; Sinha et al., 2023; Park et al., 2025; Habimana et al., 2020). The pathophysiology of sepsis is underpinned by a complex interplay of immune activation, endothelial dysfunction, and profound metabolic and mitochondrial dysregulation. Initially, sepsis is driven by the recognition of pathogen-associated molecular patterns (PAMPs) and damage-associated molecular patterns (DAMPs), which activate innate immune responses through pattern recognition receptors (Zhang et al., 2010; Li et al., 2023a). This leads to the release of pro-inflammatory cytokines such as TNF-α, IL-6, and IL-1β. While this inflammatory cascade is essential for microbial clearance, excessive or prolonged activation results in collateral tissue damage and immunosuppression (Giamarellos-Bourboulis et al., 2024; Lelubre and Vincent, 2018; Rocha et al., 2012). In addition, mitochondrial dysfunction drives sepsis by impairing energy production and redox balance, leading to bioenergetic failure and multi-organ damage (Zong et al., 2024) (Galley, 2011; Víctor et al., 2009). Furthermore, the imbalance between ROS production and antioxidant defences plays a dual role in sepsis. While ROS are essential for pathogen clearance, their uncontrolled accumulation exacerbates mitochondrial damage and cellular injury (Figure 2) (Joffre and Hellman, 2021; Brealey et al., 2002). This has prompted growing interest in therapeutic interventions aimed at restoring mitochondrial function and redox homeostasis. Furthermore, antioxidant therapy, particularly agents targeting mitochondria, offers a promising strategy for mitigating oxidative damage and improving outcomes in sepsis. Compounds such as melatonin, N-acetylcysteine, vitamin C, and selenium have demonstrated protective effects in preclinical and some clinical studies by enhancing mitochondrial integrity and reducing systemic inflammation (Mantzarlis et al., 2017; Nagar et al., 2018). This review comprehensively explores the recent advances in understanding the multifaceted role of oxidative stress in sepsis pathophysiology, with particular emphasis on mitochondrial dysfunction and the emerging therapeutic potential of antioxidants to restore redox balance, improve organ function, and enhance patient outcomes.

## Oxidative stress effect on sepsis pathology

**FIGURE 3:**
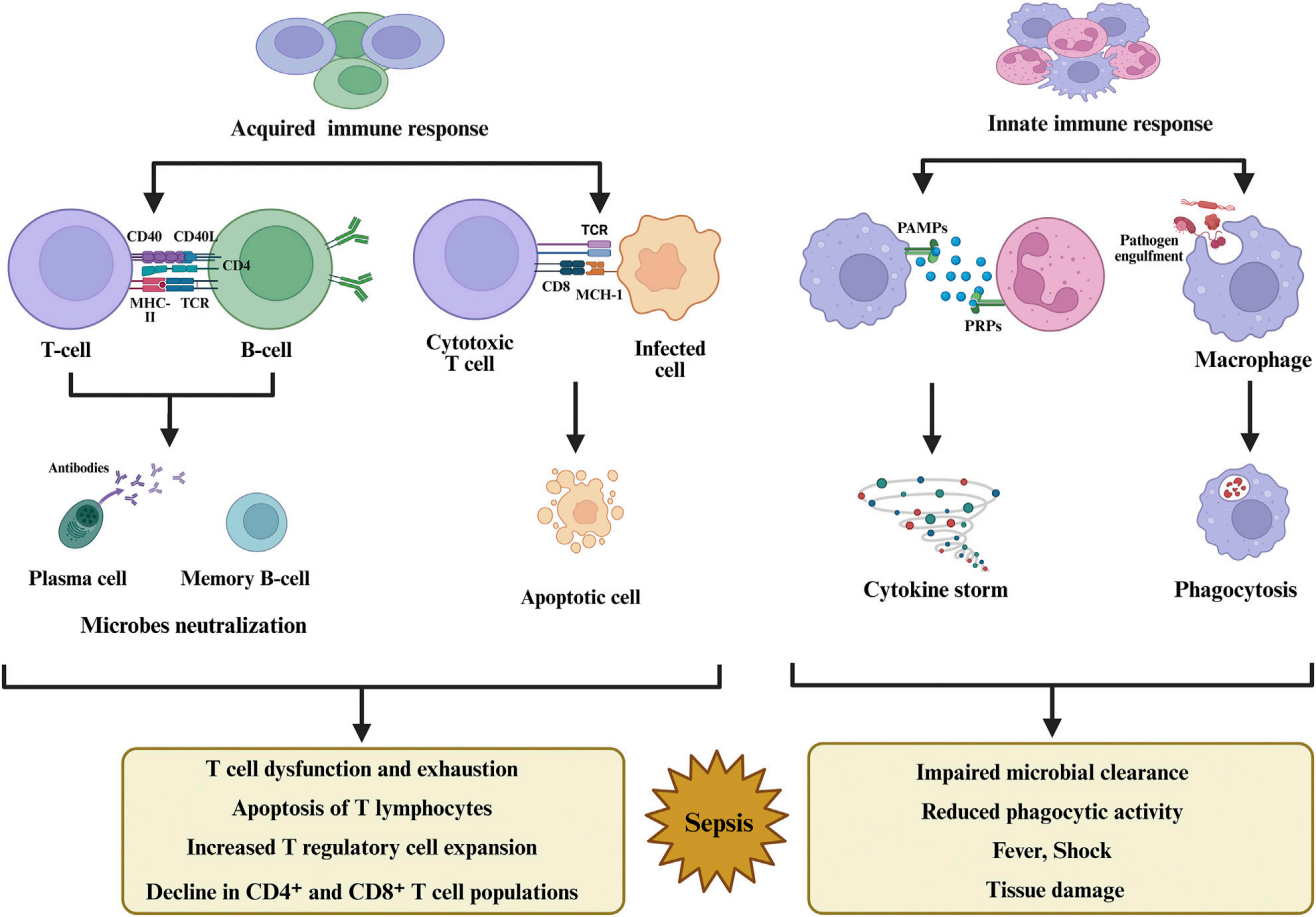
Sepsis triggers dysregulated innate and adaptive immunity, leading to inflammation, immune suppression, and organ damage.

Oxidative stress plays an essential role in the pathogenesis and progression of sepsis, primarily through the overproduction of ROS and impairment of antioxidant defence mechanisms. At the systemic level, oxidative stress contributes to vascular and endothelial dysfunction, promoting capillary leakage, microcirculatory derangements, and thrombosis. These effects impair oxygen and nutrient delivery to tissues, triggering hypoxia, metabolic acidosis, and organ failure (Higashi et al., 2014; Incalza et al., 2018). Studies have shown that oxidative stress induces endothelial cell apoptosis and disrupts the endothelial glycocalyx, facilitating leukocyte adhesion and extravasation, which worsens tissue damage (Joffre and Hellman, 2021). The combined impact on vascular tone, coagulation, and oxygen utilization is a major contributor to the development of shock and multi-organ dysfunction syndrome. During sepsis, immune cells such as neutrophils and macrophages are rapidly activated in response to microbial invasion, releasing large amounts of ROS, including superoxide anion (O_2_^−^), hydrogen peroxide (H_2_O_2_), and hydroxyl radicals (OH^−^), as part of the host’s antimicrobial arsenal (Di Meo et al., 2016). While ROS are essential for pathogen clearance, excessive and unregulated production leads to widespread cellular and tissue injury (Figure 3) (Mantzarlis et al., 2017). However, research has also revealed that free radicals function as damaging agents and as critical mediators of intracellular signaling (Chandimali et al., 2025). Broadly, oxidants include a range of primary oxygen- and nitrogen-derived species that readily interact with biomolecules, inducing oxidative modifications to lipids, proteins, and nucleic acids (Juan et al., 2021). This pathological oxidative burden is closely linked to mitochondrial dysfunction. Mitochondria are both a source and target of ROS. Under physiological conditions, the, ETC tightly regulates ROS generation. However, during sepsis, ETC dysfunction leads to electron leakage and disproportionate ROS formation, resulting in the collapse of mitochondrial membrane potential, impaired ATP synthesis, and activation of mitochondrial-mediated apoptosis (Nagar et al., 2018). Cells with depleted ATP reserves are more vulnerable to metabolic or chemical stress and consequently progress to necrotic cell death. In apoptosis, the opening of the mitochondrial permeability transition pore (MPTP), which is mediated by oxidative stress, triggers a cascade of events, including Ca^2+^ efflux, collapse of mitochondrial membrane potential, enhanced ROS formation, and the release of cytochrome c along with other pro-apoptotic molecules into the cytoplasm (Bernardi et al., 2023; Giorgi et al., 2012).

Furthermore, nitric oxide (NO) serves multiple physiological functions, acting as a neurotransmitter in the nervous system, preserving vascular homeostasis, and regulating immune responses (Andrabi et al., 2023). In addition, it contributes to intracellular signaling, gene expression, and the regulation of cellular growth and differentiation (Tuteja et al., 2004; Lundberg and Weitzberg, 2022). Sepsis triggers the induction of inducible nitric oxide synthase (iNOS), resulting in excessive and sustained NO release. Excessive NO production interacts with ROS to generate peroxynitrite (ONOO^−^), a highly reactive nitrogen species capable of nitrating proteins and inactivating mitochondrial enzymes (Takatani et al., 2018; Cinelli et al., 2020). The resulting damage intensifies cellular dysfunction and suppresses mitochondrial respiration, thereby aggravating the bioenergetic failure that underlies severe sepsis (Víctor et al., 2009; Takatani et al., 2018). Pathogen-associated molecules such as lipopolysaccharide (LPS) play a critical upstream role in the oxidative cascade. In addition, the binding of LPS to the Toll-like receptor 4 (TLR4) complex activates NADPH oxidase and promotes mitochondrial ROS generation, while simultaneously inducing iNOS expression (Galley, 2011; Luo et al., 2025). This integrates microbial recognition with redox imbalance, ensuring both antimicrobial defense and collateral oxidative injury. At the same time, ROS and RNS act as signaling mediators that activate nuclear factor kappa B (NF-κB), a transcription factor central to the inflammatory response. Through ROS-mediated phosphorylation of IκB kinase, NF-κB is released from its inhibitor and translocates to the nucleus, where it induces transcription of pro-inflammatory cytokines, chemokines, adhesion molecules, and iNOS itself. This promotes further oxidant production and cytokine release, perpetuating systemic inflammation and tissue damage (Sun, 2017; Lawrence, 2009). Tsukamoto et al. found that LPS engagement with lipopolysaccharide-binding protein (LBP) and CD14 initiates TLR4 signaling that drives oxidative cascades through excessive ROS production (Tuteja et al., 2004). A study showed that LPS administration caused a strong, dose- and time-dependent activation of NF-κB across multiple organs, as demonstrated in transgenic NF-κB reporter mice. The activation was associated with significant IκB degradation and nuclear translocation of NF-κB, resulting in increased transcription of pro-inflammatory mediators (Blackwell et al., 2000). The outcome was a robust rise in cytokines such as TNF-α, IL-6, KC, MIP-2, and MCP-1, which promoted neutrophil infiltration and widespread inflammatory injury. Importantly, when NF-κB signaling was inhibited using a dominant-negative IκB mutant, both cytokine levels and tissue damage were markedly reduced, confirming that ROS-mediated NF-κB activation directly drives cytokine overproduction and multi-organ dysfunction in sepsis (Blackwell et al., 2000). Another study presented that NF-κB activity in peripheral blood mononuclear cells was significantly higher in patients with severe sepsis who did not survive compared to survivors, and strongly correlated with illness severity (APACHE II score) (Arnalich et al., 2000). Furthermore, elevated NF-κB activity was linked with increased plasma levels of IL-6, while survivors displayed higher concentrations of anti-inflammatory mediators such as IL-1 receptor antagonist (IL-1ra) and IL-10 (Arnalich et al., 2000). Moreover, Metnitz et al. showed that in sepsis-related ARDS, patients had depleted antioxidant defenses with persistently high malondialdehyde (MDA) levels, indicating ongoing lipid peroxidation. Elevated myeloperoxidase (MPO) activity in bronchoalveolar fluid reflected neutrophil-driven oxidative stress, contributing to sustained lung injury despite micronutrient supplementation (Metnitz et al., 1999). Nevertheless, sepsis induces the upregulation of numerous genes associated with inflammation, vascular dysfunction, and immune regulation. Classical pro-inflammatory cytokines (IL1B, IL6, TNF) and pattern recognition receptors (TLR2, TLR4) are markedly increased, along with adhesion molecules (VCAM1, ICAM1) and chemokines (CCL2, CXCL8) that promote leukocyte recruitment and vascular injury (Falcão-Holanda et al., 2021; Behairy et al., 2022; He et al., 2024; Warford et al., 2017). Furthermore, Zhang et al. reported that MMP-9 expression is upregulated in sepsis-induced acute lung injury, where it exerts a protective role by promoting the release of soluble RAGE (sRAGE). This mechanism suppresses RAGE/NF-κB signaling, thereby reducing inflammation, oxidative stress, and lung damage, ultimately improving survival (Zhang et al., 2021). Jakobsson et al. investigated the role of S100A8/A9 (calprotectin) in sepsis and its link to sepsis-induced myocardial dysfunction (SIMD). They found that plasma S100A8/A9 levels were significantly elevated in severe sepsis patients and correlated with left ventricular systolic dysfunction and higher SOFA scores. In a mouse model of endotoxemia, LPS rapidly induced S100A8/A9 release and acute cardiac depression, while genetic deficiency of S100A9 or pharmacological blockade of S100A8/A9 with ABR-238901 prevented or reversed myocardial dysfunction and reduced systemic inflammation (Jakobsson et al., 2023). Additional genes upregulated in sepsis include adrenomedullin (ADM), IRAK3, and arachidonate 5-lipoxygenase (ALOX5/5-LOX). Lundberg et al. showed that ADM levels are significantly elevated in sepsis and septic shock compared with non-septic patients. High circulating ADM was strongly associated with disease severity, organ failure, vasopressor need, and 30-day mortality, highlighting its value as both a biomarker and predictor of outcome in septic patients (Lundberg et al., 2020). Alongside, Deng et al. found that IRAK3 (IRAK-M) is markedly upregulated in sepsis, particularly in alveolar macrophages, where it suppresses TLR signaling and reduces cytokine production. This leads to impaired bacterial clearance and contributes to the immunosuppressive state of sepsis, while mice lacking IRAK3 exhibited improved host defense and survival (Deng et al., 2006). Xie et al. demonstrated that ALOX5 is markedly upregulated in sepsis, where it amplifies inflammatory and oxidative pathways contributing to septic cardiomyopathy. Increased ALOX5 expression elevated leukotriene B4 levels, promoted M1 macrophage polarization, and worsened cardiac injury, whereas ALOX5 knockout or pharmacological inhibition with zileuton reduced inflammation, preserved cardiac function, and improved survival (Xie et al., 2021). Recent evidence shows that long non-coding RNAs (lncRNAs) are critically involved in the pathogenesis and diagnosis of sepsis. Zheng et al. identified 14 lncRNA pairs (SepSigLnc) with reversed expression patterns between septic patients and healthy controls, achieving very high diagnostic accuracy (AUC >0.99 in some cohorts). These lncRNAs were enriched in pathways related to immune regulation, cell fate determination, and steroid hormone responses, linking them directly to mechanisms of sepsis progression (Zheng et al., 2021). In addition, Liao et al. reported that lncRNAs exhibit strong diagnostic and prognostic value in sepsis, with pooled AUCs of 0.88 for diagnosis and 0.84 for predicting 28-day mortality. Specific lncRNAs, such as MALAT1 and NEAT1, were consistently linked to sepsis severity and poor outcomes (Liao et al., 2022).

## Therapeutic effect of antioxidants in sepsis

### Enzyme-based antioxidants

#### Superoxide dismutase (SOD)

SOD is a key antioxidant enzyme that has a protective role in sepsis by mitigating oxidative stress through the detoxification of superoxide anions. Several studies have explored the therapeutic application of both native and mimetic forms of SOD to counteract this oxidative burden (Table 1). In septic models, SOD mimetics have demonstrated potential in limiting inflammation and improving survival outcomes by neutralizing superoxide and reducing peroxynitrite formation. This protective mechanism is particularly effective in preserving mitochondrial function and vascular responsiveness, two critical factors in the pathophysiology of sepsis-related organ failure (Salvemini and Cuzzocrea, 2003). Zainumi et al. investigated the therapeutic role of SOD in a rat model of sepsis induced by cecal ligation and puncture, a widely accepted method that mimics polymicrobial infection. The treatment markedly reduced superoxide accumulation, preserved mitochondrial respiratory function, and attenuated systemic oxidative damage. SOD administration also improved mean arterial pressure and reduced organ injury scores, particularly in the liver and kidneys (Zainumi et al., 2022). Bo et al. also investigated the protective effects of orally administered melon-derived SOD in a rat model of sepsis. Their results demonstrated that SOD supplementation significantly improved renal function by reducing serum creatinine and neutrophil gelatinase-associated lipocalin (NGAL) levels (Bo et al., 2022). Furthermore, Bao et al. foundthat in LPS-induced sepsis, neutrophils release extracellular vesicles containing mitochondrial SOD2, which play a protective role against oxidative endothelial injury and DIC. These vesicles significantly reduced ROS levels, maintained vascular barrier function, and decreased fibrin deposition. When transferred into septic or neutropenic mice, the SOD2-enriched vesicles improved tissue perfusion and survival, while inhibition of SOD2 activity abolished these benefits (Bao et al., 2022). In addition, Serena et al. study revealed that SOD1-overexpressing mice exhibited significantly higher survival rates, reduced histopathological damage in the liver and lungs, and decreased serum levels of pro-inflammatory cytokines. Additionally, SOD1 upregulation attenuated oxidative stress, as evidenced by lower MDA levels and preserved glutathione content. Experimental studies further support the use of low molecular weight SOD mimetics as promising therapeutic agents in septic shock. These compounds penetrate cells more efficiently and retain catalytic activity, offering targeted ROS neutralization in inflamed tissues (Albuszies and Brückner, 2003). For instance, melon-derived SOD formulations significantly reduced biomarkers of acute kidney injury (e.g., NGAL and serum creatinine) by restoring oxidative balance in septic mice, suggesting organ-specific therapeutic benefits (Ismy et al., 2022). Furthermore, adjunctive SOD therapy is linked to improved endothelial function and reduced expression of iNOS, thereby curbing the inflammatory cascade in sepsis (Prauchner, 2017). Coleman et al. investigated the therapeutic potential of a manganese porphyrin compound (MnTnBuOE-2-PyP^5+^), a SOD mimetic, in a mouse model of endotoxemia. Their study demonstrated that pre-treatment with this compound significantly improved survival, reduced serum cytokine levels, and mitigated liver and lung injury following LPS challenge (Coleman et al., 2017).

**TABLE 1:** Clinical and preclinical investigations of superoxide dismutase (SOD) as a biomarker and therapeutic modulator in sepsis pathophysiology.

Study	Model/Population	SOD formulation/Measurement method	Main findings and clinical/Experimental outcomes	References
Costa et al. (Clinical observational)	132 ICU septic shock patients (no AKI at baseline)	Erythrocyte SOD1 activity measured (U/mg Hb)	Lower SOD1 activity associated with septic AKI. Activity >3.32 U/mg Hb was protective (AUC 0.686, p < 0.01).	Costa et al. (2016)
Costa et al., 2018 (Clinical observational)	137 septic shock patients (SOD1 SNPs analyzed)	Erythrocyte SOD1 activity + 9 SNPs (e.g., rs2070424)	Higher SOD1 activity correlated with lower ICU mortality (OR 0.02; p = 0.037). SNP rs2070424 impacted SOD1 activity but not mortality.	Costa et al. (2018)
Guerreiro et al. (Clinical observational)	96 septic patients (APACHE II and MODS recorded)	Plasma SOD activity (Day 1 and Day 2), endogenous	Higher SOD activity correlated with worse prognosis. A ≥25% decline in SOD activity within 24 h was associated with improved survival.	Guerreiro et al. (2010)
Stanic et al. (Clinical prospective)	87 septic patients (Gram-positive vs. Gram-negative infection groups)	Endogenous plasma SOD on Day 1 and Day 2	SOD levels did not significantly differ across bacterial types or predict mortality. Limited predictive value for causative agent or outcome.	Stanic et al. (2024)
Zainumi et al. (Preclinical rat model)	LPS-induced sepsis in Wistar rats, 4 groups (n = 6/group)	Oral CME-gliadin SOD extract (1 IU/day and 5 IU/day for 28 days)	Both doses reduced TNF-α, MDA, and lactate levels significantly (p < 0.001). 5 IU/day had greater antioxidant and anti-inflammatory effects than 1 IU/day.	Zainumi et al. (2022)
Bao et al. (Preclinical mouse model)	LPS-induced DIC model; adoptive neutrophil transfer	Mitochondrial SOD2 in neutrophil-derived extracellular vesicles	SOD2-containing vesicles prevented endothelial ROS accumulation and DIC, improving survival. Demonstrated a protective neutrophil-endothelium interaction in sepsis.	Bao et al. (2022)

#### Catalase

Catalase (CAT), a heme-containing enzyme, has a critical role in cellular redox homeostasis by catalyzing the decomposition of H_2_O_2_ into water and molecular oxygen, thereby mitigating oxidative stress. Several studies have explored its therapeutic application in experimental models of sepsis (Table 2). For instance, Li et al. developed a PEGylated form of catalase (CAT-PEG) to enhance its stability and bioavailability *in vivo*. In a murine model of sepsis, treatment with CAT-PEG significantly reduced systemic inflammation and improved survival outcomes, underscoring its potential as a viable antioxidant therapy in septic conditions (Li et al., 2021). Furthermore, comprehensive reviews of antioxidant therapies have emphasized the relevance of catalase and other ROS-scavenging enzymes in mitigating the molecular cascades triggered by oxidative and nitrosative stress during sepsis (Kumar et al., 2022; Victor et al., 2005). Che et al. developed a multifunctional polyphenol-copper nanozyme that mimics both catalase and superoxide dismutase activity. This nanozyme effectively suppressed endoplasmic reticulum stress, reduced lipid peroxidation, and improved hepatocellular architecture, demonstrating potent systemic protection (Che et al., 2025). Catalase upregulation appears to correlate with improved outcomes in various sepsis-associated organ injuries. Xia et al. demonstrated that the small molecule anemonin increased catalase expression in lung tissue, thereby reducing oxidative damage and inflammation in sepsis-induced acute lung injury *via* modulation of the NF-κB and Nrf2 pathways (Xia et al., 2025). Complementary data from Kumar et al. showed that catalase expression was significantly upregulated in rats treated with a dispiro-indanedione-parthenin hybrid compound, coinciding with suppression of MAPK/NF-κB signaling and improved redox status in septic tissues (Kumar et al., 2025). Interestingly, therapies that indirectly modulate catalase also confer benefit. Abdelmawgood et al. utilized chrysin-loaded PLGA nanoparticles to elevate catalase and GPx activity in the spleen of septic mice, reducing lipid peroxidation and modulating immune cell responses (Abdelmawgood et al., 2025). Moreover, Wang et al. found that mitochondrial dysfunction in sepsis-induced endothelial damage was associated with suppressed catalase activity. Pharmacological interventions that restored catalase levels helped reestablish mitochondrial integrity and barrier function in endothelial cells (Wang S. et al., 2025). Furthermore, Erel et al. reported increased hepatic catalase levels following ozone therapy in a rat model of cecal perforation-induced sepsis, suggesting catalase as both a therapeutic effector and a biomarker of response (Erel et al., 2024). Collectively, these studies underscore catalase as a multifaceted therapeutic agent in sepsis. Whether through direct enzyme delivery, gene regulation, or catalytic mimetics, catalase-targeted strategies effectively dampen oxidative injury, suppress inflammation, and enhance host resilience against septic insult.

**TABLE 2:** Clinical and preclinical studies on catalase and glutathione activity and effects in sepsis.

Study	Study type/Model	Intervention	Outcome	References
Leff et al.	Clinical study (septic patients with/without ARDS)	Endogenous serum catalase measured (no external dose)	Increased catalase in septic ARDS patients; correlated with H_2_O_2_ scavenging and endothelial protection.	Leff et al. (1992)
Li et al.	Preclinical (LPS/D-GalN-induced sepsis in mice)	PEGylated catalase (CAT-PEG, IV, single dose)	Reduced TNF-α, IL-6, AST/ALT, and mortality; improved survival and antioxidant defense.	Li et al. (2021)
Abdelmawgood et al.	Preclinical (LPS-induced sepsis in C57BL/6 mice)	Chrysin-loaded PLGA nanoparticles (CHR-NP, 50 mg/kg × 6 days + LPS 10 mg/kg i.p.)	Elevated catalase and GPx; decreased oxidative stress and splenic inflammation.	Abdelmawgood et al. (2025)
Kumar et al.	Preclinical (LPS-induced sepsis and macrophages)	Dispiro-indanedione hybrid of parthenin (DIHP, 1.25–5 μM *in vitro*)	Upregulated catalase and Nrf2; suppressed NF-κB/MAPK and inflammatory cytokines.	Kumar et al. (2025)
Ortolani et al.	Clinical [patients with early septic shock (n = 30)]	Intravenous GSH 70 mg/kg/day ± NAC 75 mg/kg/day for 5 days	Exogenous GSH restored redox balance, reduced lipid peroxidation, and improved clinical status.	Ortolani et al. (2000)
Babu et al.	Preclinical [neonatal rat hepatocytes exposed to H_2_O_2_/NO (sepsis mediators)]	Exogenous GSH (10 mmol/L)	GSH reversed oxidative inhibition of hepatocyte metabolism and improved mitochondrial activity.	Babu et al. (2001)
Wang et al.	Preclinical (CLP-induced sepsis in mice; LPS-treated cardiomyocytes)	Endogenous GSH modulation *via* VDAC3/DHODH pathway	Sepsis reduced myocardial GSH; restoring GSH alleviated ferroptosis and cardiac injury.	Wang et al. (2025b)
Tang et al.	Preclinical (LPS and CLP sepsis models in mice; cardiomyocytes *in vitro*)	Narciclasine 0.1 mg/kg (i.p., 3 days)	Narciclasine prevented GSH depletion, enhanced GPX4 activity, and protected mitochondria.	Tang et al. (2025)
Mileva et al.	Oxidative-stress model (influenza-induced systemic inflammation in mice)	S-Adenosyl-L-methionine 100 mg/kg (GSH precursor)	SAM increased hepatic GSH synthesis and reduced oxidative liver damage.	Mileva et al. (2022)

#### Glutathione

Glutathione (GSH), a tripeptide composed of glutamate, cysteine, and glycine, serves as a critical intracellular antioxidant that regulates redox homeostasis, detoxifies ROS, and modulates immune responses (Narayanankutty et al., 2019). In the context of sepsis, GSH plays a multifaceted role in counteracting the oxidative stress and mitochondrial dysfunction that underlie organ failure and systemic inflammation. During sepsis, both the synthesis and bioavailability of GSH are significantly impaired, leading to uncontrolled ROS accumulation and cellular injury (Table 2). Biolo et al. study highlighted the dysregulation of glutathione metabolism in septic patients and emphasized that the impaired biosynthetic response may contribute to heightened oxidative damage and worsened clinical outcomes (Biolo et al., 2007). GSH has shown potential as a direct antioxidant and as an adjunctive therapy. A study by Liu et al. investigated the use of glutathione-assisted continuous renal replacement therapy (CRRT) in septic patients with acute kidney injury. This combination improved peripheral receptor expression and mitigated systemic inflammatory responses, suggesting synergistic effects between GSH and extracorporeal blood purification (Liu et al., 2024). Additionally, Ortolani et al. found that administration of GSH and its precursor NAC reduced lipid peroxidation and oxidative tissue damage in patients with early septic shock, affirming the therapeutic viability of antioxidant co-therapy in acute care settings (Ortolani et al., 2000). In addition, Mileva et al. showed that combining oseltamivir with SAM, a glutathione precursor, reduced oxidative stress during influenza by lowering MDA levels, restoring GSH, normalizing antioxidant enzymes, and improving survival (Mileva et al., 2022). Furthermore, Tang et al. demonstrated that restoring GSH levels protects against sepsis-induced cardiac dysfunction by inhibiting BNIP3-mediated mitophagy and ferroptosis, preserving mitochondrial function, and reducing lipid peroxidation. Enhancing GSH, *via* direct supplementation or activation of SLC7A11 and GPX4, also suppressed ferroptosis and myocardial injury in sepsis models. (Tang et al., 2025). Further evidence by Wang et al. highlighted glutathione’s role in mitochondrial regulation and inhibition of oxidative damage in septic myocardial tissue. The study identified a regulatory axis (VDAC3/DHODH) that enhances GSH-mediated ferroptosis resistance, demonstrating significant cardioprotective outcomes *in vivo* (Wang J. et al., 2025). Nevertheless, Jin et al. found that restoring GSH levels alleviated hepatic ferroptosis and oxidative stress by upregulating PI3K/Akt/Nrf2 signaling and enhancing the SLC7A11/GPX4 axis. This cascade effectively prevented mitochondrial membrane potential collapse and hepatocyte apoptosis (Yin Y. et al., 2025). Experimental data also support the hepatoprotective effects of GSH. Babu et al. found that glutamine and its dipeptides restored oxidative metabolism in neonatal hepatocytes impaired by septic mediators (H_2_O_2_, NO), likely through boosting glutathione synthesis rather than the Krebs cycle. Blocking glutathione synthesis abolished the effect, while exogenous glutathione replicated it, confirming the mechanism (Babu et al., 2001). Additionally, Zhao et al. utilized NAD+ -loaded neutrophils to activate antioxidant pathways, including GSH-related enzymes. The resulting downregulation of TLR4/NF-κB/NLRP3 signaling led to suppressed cytokine storms and enhanced organ protection in septic mice (Zhao et al., 2025). Li et al. discussed glutathione synthesis as a key determinant in mitigating cuproptosis and disulfidptosis, two novel, metal-dependent cell death pathways exacerbated in sepsis. By regulating mitochondrial ion homeostasis and redox-sensitive protein folding, glutathione supports cytoprotective responses under extreme oxidative burden (Li et al., 2025). Moreover, in a prospective study, reduced glutathione administered alongside blood purification therapy significantly decreased endotoxin levels, inflammatory mediators, and hepatic injury in septic shock patients (Liu and Meng, 2024).

### Vitamin-based antioxidants

#### Vitamin A

Vitamin A is a fat-soluble micronutrient with immunomodulatory and antioxidant properties, and has a significant role in the regulation of innate and adaptive immune responses. In sepsis, vitamin A was investigated for its therapeutic potential due to its influence on epithelial barrier integrity, immune regulation, and oxidative stress mitigation (Table 3) (Willinger, 2019). One of the earliest mechanistic links between vitamin A and sepsis lies in its capacity to modulate inflammatory responses through its active metabolite, retinoic acid. In an experimental rodent model, Carlson et al. demonstrated that antioxidant vitamin therapy, including vitamin A at 417 U/kg, ameliorated myocardial apoptotic activity and reduced inflammatory signaling pathways associated with septic pathophysiology (Carlson et al., 2006). Vitamin A has been implicated in maintaining the integrity of epithelial and endothelial barriers, especially within the gastrointestinal tract (Pham et al., 2021). This is of particular importance, as gut barrier dysfunction is a recognized driver of systemic infection and endotoxemia in septic patients. Retinoic acid is found to enhance tight junction protein expression and reduce bacterial translocation in preclinical sepsis models, potentially mitigating multi-organ dysfunction (Martinez Ramos et al., 2025). However, the therapeutic efficacy of vitamin A in human sepsis remains contentious. A prospective, double-blind, placebo-controlled trial by Cherukuri et al. assessed high-dose vitamin A administration in septic adult patients, nearly half of whom were vitamin A deficient at baseline. The intervention failed to demonstrate significant improvements in outcomes compared to placebo, suggesting that supplementation alone may be insufficient without addressing broader metabolic and inflammatory dysfunctions inherent to sepsis (Cherukuri et al., 2019). In pediatric populations, emerging evidence is more promising. A recent randomized controlled trial investigated vitamin A supplementation in children with sepsis and reported improvements in clinical parameters, including shortened ICU stays and reduced inflammatory markers, indicating potential age-dependent therapeutic responsiveness (Fu et al., 2025). Moreover, epidemiological data support a high prevalence of vitamin A deficiency in septic patients. A study by Zhang et al. revealed a significant association between low serum retinol levels and illness severity in critically ill children with sepsis, highlighting the importance of baseline vitamin A status in determining treatment responsiveness (Zha et al., 2019). Garg et al. examined the impact of vitamin A supplementation on bronchopulmonary dysplasia (BPD) in extremely low birth weight neonates. Their findings showed a statistically significant reduction in BPD at 36 weeks postmenstrual age and a borderline significant decrease in the combined outcome of mortality or BPD. However, no significant benefit was observed at 28 days of life. While the results suggest potential benefit (Garg et al., 2019). Moreover, a randomized, double-blind, placebo-controlled trial evaluated the effect of weekly low-dose vitamin A supplementation (10,000 IU) on immune responses in pregnant and lactating Ghanaian women. Vitamin A significantly increased the ratio of proinflammatory (IFN-γ, TNF-α) to anti-inflammatory (IL-10) cytokines during pregnancy and postpartum, indicating a shift toward Th1-type immune responses. Clinically, this modulation suggests vitamin A may enhance resistance to intracellular infections such as malaria without adverse pregnancy outcomes when given in low doses (Binka et al., 1995).

**TABLE 3:** Summary of major clinical studies evaluating vitamins therapy in sepsis.

Study	Study model/Participants	Regimen	Main outcomes	References
Fowler et al.	Randomized controlled trial (24 patients suffering from severe sepsis; 8 received low-dose, 8 high-dose, and 8 placebo)	Low dose = 50 mg/kg/day or high dose = 200 mg/kg/day IV over 96 h	Significant reduction in SOFA, CRP, and PCT, indicating better organ and inflammatory control.	Fowler et al. (2014)
Zabet et al.	Randomized controlled trial [28 surgical ICU patients with septic shock on vasopressor therapy (14 per group)]	25 mg/kg IV every 6 h for 72 h *versus* placebo	Mortality 14.3% vs. 64.3% (*p = 0.009*); lower vasopressor use and faster stabilization.	Zabet et al. (2016)
Fowler et al.	Randomized controlled trial [167 patients with sepsis-associated ARDS (84 intervention, 83 placebo)]	50 mg/kg IV every 6 h for 96 h compared with placebo	Mortality 29.8% vs. 46.3% (*p = 0.03*); mSOFA improvement.	Fowler et al. (2019)
Ahn et al.	Retrospective (75 ICU patients with severe sepsis or septic shock under mechanical ventilation (35 treated, 40 controls)]	2 g IV every 8 h until ICU discharge vs. standard therapy	No significant change in SOFA, mortality, or shock reversal.	Ahn et al. (2019)
Nakajima et al.	Retrospective (785 patients with severe burns (burn index ≥15); 157 received Vitamin C, 628 controls)	Vitamin C dosage ≥10 g to <24 g or ≥24 g IV	Mortality 45.9% vs. 58.0% (p = 0.006); no extra benefit with ≥24 g.	Nakajima et al. (2019)
Moskowitz et al.	Randomized controlled trial [205 patients with septic shock (103 treatment/102 placebo)]	Hydrocortisone 50 mg, Vitamin C 1.5 g, and Thiamine 100 mg IV every 6 h for 4 days	No significant difference in SOFA score, kidney injury, or 30-day mortality.	Moskowitz et al. (2020)
Sevransky et al.	Randomized controlled trial [501 patients with septic shock (252 treatment/249 placebo)]	Hydrocortisone 50 mg, Vitamin C 1.5 g, and Thiamine 100 mg IV every 6 h for 4 days	No increase in ventilator- or vasopressor-free days; trial ended early, underpowered for benefit.	Sevransky et al. (2021)
Wald et al. et al.	Retrospective (557 septic shock with vasopressors [47 HAT/181 HCT/333 control)]	Hydrocortisone 50 mg/m^2^ q6 h, Vitamin C 30 mg/kg q6 h × 4 days (up to 1,500 mg/dose), Thiamine 4 mg/kg/day divided BID	Lower mortality vs. control at 30 days (9% vs. 28%) and 90 days (14% vs. 35%).	Wald et al. (2020)
Carlson et	Preclinical (Sprague-Dawley rats with *S. pneumoniae*-induced sepsis)	Oral vitamin A (417 U/kg) + C + E + Zn, twice daily × 3 days pre-infection	Vitamin A improved myocardial antioxidant defense, reduced cytokine-induced apoptosis, and preserved cardiac function.	Carlson et al. (2006)
Lavanya et al.	Clinical [adults with severe sepsis (n = 64)]	Intramuscular vitamin A 100,000 IU daily × 7 days vs. placebo	Vitamin A deficiency common (≈50%); supplementation slightly shortened ICU stay but showed no mortality benefit.	Cherukuri et al. (2019)
Fu et al.	Clinical [children with sepsis (n = 156, RCT)]	Age-adjusted oral vitamin A (5,000–20,000 IU) × 3 days vs. placebo	Vitamin A lowered lactate and procalcitonin, enhanced clearance, and correlated with higher albumin; safe and well tolerated.	Fu et al. (2025)
Carlson et al.	Preclinical (rodent sepsis model)	Oral vitamin A with C/E/Zn pretreatment	Vitamin A stabilized endothelial integrity, improved immune response, and limited oxidative injury in septic tissue.	Carlson et al. (2006)
Wang et al.	Preclinical (CLP-induced sepsis in mice; sera from sepsis/ARDS patients)	α-Tocopherol 100 mg/kg i.p. ± MUC1 modulation	Reduced ferroptosis and oxidative stress *via* Nrf2–GPX4; improved lung injury.	Wang et al. (2022a)
Dang et al.	Clinical (182 critically ill children)	Serum vitamin E measured at admission	Deficiency strongly associated with septic shock and higher mortality.	Dang et al. (2021)
Atli et al.	Preclinical (rat fecal peritonitis model)	α-Tocopherol 300 mg/kg/day × 3 days (± vit C 500 mg/kg/day)	Lower TNF-α, IL-6, CRP, MDA; improved lung histology and CT findings.	Atli et al. (2012)
Bajčetić et al.	Clinical [preterm neonates with sepsis (n = 31)]	Oral vitamin E 25 IU/day × 60 days vs. none	Altered antioxidant enzyme profile; no clear clinical benefit.	Bajčetić et al. (2014)
Canbolat et al.	Preclinical (Wistar rats with fecal peritonitis-induced sepsis)	α-Tocopherol 300 mg/kg/day × 3 days ± vit C	Decreased oxidative/inflammatory markers and improved lung protection.	Canbolat et al. (2022)

#### Vitamin C

Vitamin C has emerged as a compelling adjunctive therapy in the management of sepsis, driven by its antioxidant, anti-inflammatory, and endothelial-stabilizing properties. Under intense oxidative stress and excessive ROS production, the requirement for vitamin C increases significantly as somatic and immune cells consume greater amounts of this antioxidant; moreover, the heightened proliferation and turnover of leukocytes amplify its depletion, since these cells can concentrate vitamin C up to a hundredfold higher than plasma levels, ultimately leading to a substantial reduction in systemic vitamin C availability during sepsis (Oudemans-van Straaten et al., 2014; Wilson, 2013). Moreover, vitamin C enhances neutrophil and lymphocyte activity, limits neutrophil necrosis and extracellular trap formation, and modulates nuclear responses to hypoxia and stress by regulating HIF-1α and inducing NF-κB-related epigenetic changes (Fisher et al., 2011; Carr and Maggini, 2017). Vitamin C deficiency is frequently observed in critically ill septic patients, and repletion strategies, particularly *via* high-dose intravenous vitamin C (HDIVC), have garnered significant scientific interest (Table 3) (Marik, 2018). Kashiouris et al. found that HDIVC was associated with improved organ function and a trend toward reduced mortality in septic patients (Kashiouris et al., 2020). A meta-analysis by Liang et al. further refined these findings, demonstrating that intravenous vitamin C significantly reduced vasopressor requirements and improved the Sequential Organ Failure Assessment (SOFA) score (Liang et al., 2023). Phase I randomized, double-blind, placebo-controlled clinical trial investigated intravenous vitamin C in 24 ICU patients with severe sepsis. Patients received placebo, low-dose (50 mg/kg/day), or high-dose (200 mg/kg/day) vitamin C every 6 hours for 4 days. Both doses were safe and well tolerated, but the high-dose regimen achieved markedly higher plasma concentrations and produced a faster, greater reduction in SOFA scores and inflammatory biomarkers (CRP and procalcitonin), while preventing thrombomodulin elevation (Fowler et al., 2014). A double-blind randomized clinical trial evaluated the effect of HDIVC on vasopressor requirements in 28 surgical ICU patients with septic shock. Patients received either vitamin C (25 mg/kg every 6 h for 72 h) or a placebo. Vitamin C administration significantly reduced the mean dose and duration of norepinephrine use and markedly lowered 28-day mortality (14.3% vs. 64.3%), with no adverse effects observed (Zabet et al., 2016). In addition, a sepsis with severe respiratory failure clinical trial by Fowler et al. identified that HDIVC did not significantly improve the modified SOFA score at 96 h but did reduce 28-day mortality (29.8% vs. 46.3%) and lower inflammatory and endothelial injury markers, including CRP and thrombomodulin (Fowler et al., 2019). Plasma syndecan-1, a marker of endothelial glycocalyx injury and a predictor of mortality in severe sepsis and acute respiratory distress syndrome, was significantly reduced following HDIVC treatment (Kashiouris et al., 2019). Furthermore, intravenous vitamin C was shown to reduce inflammatory and cardiac injury markers and lower SOFA scores, indicating enhanced inflammation control and cardioprotective effects (Jiang et al., 2024). In septic ICU patients with elevated lactate, treatment with vitamin C and thiamine lowered hospital mortality, showing the strongest benefit when both were given together, especially in vasopressor-dependent cases (Byerly et al., 2020). Marik et al. reported that combining hydrocortisone, vitamin C, and thiamine in patients with severe sepsis and septic shock significantly reduced hospital mortality (8.5% vs. 40.4%) and improved organ function (Marik et al., 2017).

#### Vitamin E

Vitamin E, a fat-soluble antioxidant best known for its α-tocopherol isoform, serves a vital function in shielding cell membranes from oxidative injury. In the context of sepsis, vitamin E was investigated for its ability to suppress inflammatory responses, preserve cellular integrity, and potentially improve clinical outcomes (Table 3). Pein et al. revealed that endogenous metabolites of vitamin E, particularly α-13′-COOH, exert anti-inflammatory effects by directly inhibiting 5-lipoxygenase (5-LO), a key enzyme in leukotriene biosynthesis. These metabolites reduced inflammatory lipid mediator production in human neutrophils and monocytes, with potency comparable to pharmaceutical 5-LO inhibitors (Pein et al., 2018). Minter et al. showed that MitoVitE, α-tocopherol, and Trolox all reduced oxidative stress, NFκB activation, and cytokine release in endothelial cells under sepsis-like conditions. MitoVitE provided the strongest effect by preserving mitochondrial membrane potential and broadly downregulating TLR2/4 pathway genes, highlighting the added benefit of mitochondrial targeting (Minter et al., 2020). Atli et al. investigated the protective effects of vitamin E and selenium against sepsis-induced lung injury in a rat model (Atli et al., 2012). Both antioxidants, administered individually or in combination, significantly improved blood gas parameters, reduced leukocyte and CRP levels, and elevated glutathione peroxidase activity compared to untreated septic rats. Histopathological analysis revealed decreased inflammation, congestion, edema, and emphysema in the lung tissues of treated groups. Mechanistically, vitamin E stabilizes cell membranes by neutralizing lipid peroxides (Atli et al., 2012). Similarly, Durant et al. observed that vitamin E reduced superoxide anion overproduction and improved microcirculatory flow, highlighting its role in redox regulation during septic states (Durant et al., 2004). Canbolat et al. showed that vitamin E significantly reduced sepsis-induced lung injury in a rat model by lowering inflammatory cytokines and MDA. It improved lung histopathology and gas exchange, likely through its antioxidant action and membrane-stabilizing effects, which help preserve alveolar-capillary integrity (Canbolat et al., 2022). Furthermore, Wang et al. demonstrated that Mucin one enhances the protective effect of vitamin E against sepsis-induced acute lung injury by inhibiting ferroptosis *via* the GSK3β/Keap1-Nrf2-GPX4 pathway. Vitamin E reduced oxidative stress and lipid peroxidation, with its efficacy significantly amplified by MUC1, suggesting a synergistic therapeutic potential (Wang Y. M. et al., 2022). Furthermore, vitamin E not only suppresses oxidative damage but also enhances endothelial NO bioavailability, thus improving vascular tone and preventing sepsis-related organ hypoperfusion. Novel delivery approaches, such as nano-emulsion formulations of Vitamin E, have been developed to optimize bioavailability, potentially broadening its therapeutic applications in sepsis and systemic inflammatory conditions (Yin W. et al., 2025). In pediatric populations, vitamin E deficiency appears common among children with sepsis and septic shock. Dang et al. reported a strong association between vitamin E deficiency and sepsis severity, proposing that correcting this deficit could support antioxidant defense mechanisms during critical illness (Dang et al., 2021). Adult-focused research has also gained traction. A retrospective cohort study by He et al. suggested that standalone vitamin E supplementation in ICU patients with sepsis was associated with a reduction in 28-day mortality (He et al., 2025). Furthermore, Bajčetić et al. studied preterm neonates with sepsis and found that while vitamin E supplementation improved glutathione peroxidase activity, it also suppressed glutathione reductase, potentially impairing redox balance. These changes may increase oxidative stress and infection risk, suggesting caution with vitamin E use in this vulnerable population (Bajčetić et al., 2014). Vitamin E has also been investigated as part of combination regimens. Wald et al. noted that vitamin E, alongside vitamins A and C, has been included in therapeutic protocols aiming to attenuate inflammatory damage and support organ function in septic patients (Wald et al., 2022). Kono et al. evaluated a novel water-soluble vitamin E derivative, E-Ant-S-GS, in a rat model of peritonitis-induced sepsis. The compound, combining vitamin E, glutathione, anthranilic acid, and succinic acid, significantly reduced IL-6 levels, neutrophil infiltration, and lung injury. Mechanistically, E-Ant-S-GS suppressed the expression of HMGB1 and PAR1, key mediators of inflammation and endothelial damage, thereby attenuating systemic inflammation and acute lung injury (Kono et al., 2012).

### Novel and adjunctive antioxidant therapies

#### N-acetylcysteine (NAC)

NAC, a potent thiol-containing antioxidant, has garnered increasing attention for its therapeutic potential in mitigating the pathophysiological consequences of sepsis, particularly through modulation of oxidative stress and inflammation. Several studies support its efficacy in various organ systems affected during sepsis. NAC inhibits NF-κB activation and upregulates nuclear factor erythroid 2-related factor 2 (Nrf2), promoting cellular antioxidant defences (Table 4) (Chen et al., 2025). Additionally, Sainglers et al. found that NAC’s inhibition of neutrophil extracellular traps, which are implicated in sepsis-related tissue injury and organ failure (Sainglers et al., 2025). Le et al. demonstrated that NAC administration significantly attenuated acute lung injury in septic rats by reducing oxidative stress, inflammatory cytokine production, and apoptosis (Le et al., 2022). Similarly, Fan et al. reported that NAC pretreatment conferred protection against sepsis-induced acute kidney injury by alleviating inflammatory damage and oxidative insult in renal tissues (Fan et al., 2020). Furthermore, Oliva et al. showed that adjunctive NAC therapy in ICU patients with septic shock due to carbapenem-resistant infections reduced 30-day mortality, emphasizing its clinical value in high-risk patient populations (Oliva et al., 2021). An *in vivo* study included a rat model, found that NAC effectively prevented pulmonary edema and acute kidney injury during sepsis, particularly under mechanical ventilation stress, by preserving mitochondrial function and capillary integrity (Campos et al., 2012). Furthermore, Liu et al. investigated the impact of NAC on von Willebrand factor (vWF) release during septic conditions. Their findings suggested that NAC disrupts vWF multimers *via* disulfide bond reduction, reducing platelet aggregation and microthrombi formation, which are central to sepsis-induced organ failure (Liu et al., 2025). Clinical translation is evidenced by Fan et al., who explored NAC as a co-therapy with low molecular weight heparin, observing significant improvements in vascular function and inflammatory modulation in septic cardiovascular syndromes (Fan et al., 2024). NAC also demonstrates immunomodulatory activity. Yang et al. showed that NAC downregulated pro-inflammatory cytokines and restored macrophage function, aiding in pathogen clearance and limiting host tissue damage (Yang et al., 2025). Similarly, Sui et al. reported that NAC enhanced the efficacy of phage-antibiotic therapy against *Pseudomonas aeruginosa and Klebsiella pneumoniae*, highlighting its role in disrupting biofilms and restoring antibiotic susceptibility (Sui et al., 2025). A clinical study found that NAC, given at 600 mg every 12 h for 5 days as an adjuvant therapy in septic shock, significantly improved outcomes. NAC reduced procalcitonin levels, lowered the SOFA score by 42%–50%, and enhanced total antioxidant capacity by increasing GSH and glutathione peroxidase (GPx) activity (Aisa-Álvarez et al., 2023). Although results from randomized clinical trials remain limited, existing animal and translational studies strongly support the adjunctive use of NAC in early-phase sepsis to prevent multiorgan failure, improve microvascular perfusion, and reduce mortality.

**TABLE 4:** Clinical and preclinical studies of novel and adjunctive antioxidant therapies.

Study	Study model/Participants	Regimen	Main outcomes (antioxidant/Protective effects)	References
Campos et al.	Preclinical (CLP-induced sepsis in rats with mechanical ventilation)	NAC 4.8 g/L in drinking water, started 2 days before CLP and continued post-procedure	NAC reduced pulmonary edema, oxidative stress, and acute kidney injury; improved lung mechanics and survival.	Campos et al. (2012)
Oliva et al.	Clinical (83 ICU patients with septic shock due to carbapenem-resistant pathogens)	NAC i.v. (dose per ICU protocol) + standard antibiotics	NAC + antibiotics lowered 30-day mortality (33% vs. 57%) and improved microvascular and organ function.	Oliva et al. (2021)
Aisa-Álvarez et al.	Clinical (131 septic shock patients)	NAC orally/nasogastric 600 mg × 3 daily × 5 days (with other antioxidants arms)	NAC reduced SOFA score by 50%, decreased oxidative stress and inflammation, and enhanced antioxidant capacity.	Aisa-Álvarez et al. (2023)
Wu et al.	Preclinical (CLP-induced septic shock with MODS in rats)	Melatonin 3 mg/kg i.v. at 3, 6, 12 h post-CLP	Melatonin reduced hypotension, NO and IL-1β levels, neutrophil infiltration, and doubled survival rate.	Wu et al. (2008)
Escames et al.	Preclinical [LPS-induced sepsis in rats (young and aged)]	Melatonin 60 mg/kg i.p. post-LPS	Restored mitochondrial complex I/IV activity, decreased mtNOS and NO, and prevented mitochondrial failure.	Escames et al. (2003)
Ozkok et al.	Preclinical (LPS-induced endotoxemia in rats)	Melatonin 10 mg/kg i.p. × 3 (30 min before and 2 and 4 h after LPS)	Restored ATP and GSH levels, prevented muscle damage, and improved mitochondrial energy metabolism.	Ozkok et al. (2016)
El-Gendy et al.	Clinical [40 neonates with sepsis (case-control)]	Melatonin 20 mg single dose + antibiotics vs. antibiotics alone	Melatonin improved CRP, WBC, and clinical condition within 72 h; effective adjunct in neonatal sepsis.	El-Gendy et al. (2018)
Sahin et al.	Preclinical (CLP-induced septic shock in rats)	Carnosine 250 mg/kg i.p., 1 h post-CLP	Improved renal function, lowered MDA and cytokines, and reduced histological kidney injury.	Sahin et al. (2013)
Schmoch et al.	Translational – murine sepsis models and human data	Anserine (β-alanyl-N-methylhistidine) administration post-sepsis induction	Reduced methylglyoxal-induced endothelial damage and capillary leakage, lowering mortality.	Schmoch et al. (2025)
Kočan et al.	Clinical (65 ICU patients with sepsis/ARDS)	Sodium selenite 750 μg/24 h infusion × 6 days	Increased GPx activity, improved oxygenation index (PaO_2_/FiO_2_), especially in ARDS subgroup.	Kočan et al. (2014)
Angstwurm et al.	Clinical (238 severe sepsis/septic shock patients)	Sodium selenite 1,000 μg bolus + 1,000 μg/day × 14 days	Reduced 28-day mortality, enhanced GPx-3, and improved hemodynamic stability.	Angstwurm et al. (2007)
Hosny et al.	Clinical (75 patients with early sepsis)	Oral omega-3 (high-dose vs. low-dose) + antioxidants × 7 days	High-dose omega-3 reduced CRP, IL-6, PCT, and SOFA; shortened ICU stay and ventilation duration.	Hosny et al. (2013)
Chen et al.	Clinical (78 severe sepsis patients with GI dysfunction)	Parenteral fish oil (10 g omega-3 + 50 g soybean oil/day × 7 days)	Reduced 60-day mortality, improved T-cell function, and decreased inflammation.	Chen et al. (2017)

#### Melatonin

Melatonin, an endogenously produced indoleamine best known for regulating circadian rhythms, has attracted increasing attention for its potent antioxidant, anti-inflammatory, and immunomodulatory properties in the context of sepsis. As a highly permeable molecule capable of crossing cellular and mitochondrial membranes, melatonin effectively scavenges reactive oxygen and nitrogen species and modulates the activity of pro-inflammatory cytokines (Srinivasan et al., 2010). Pharmacologically, melatonin’s appeal lies in its wide therapeutic index, high safety profile, and low toxicity even at high doses. Colunga et al. advocate for its inclusion in sepsis protocols due to these properties and its capacity to reduce mitochondrial dysfunction and immune dysregulation, hallmarks of septic pathophysiology (Colunga Biancatelli et al., 2020; Ibrahim et al., 2024). Escames et al. explored the role of melatonin in modulating the inflammatory response induced by LPS in mice, focusing on the expression and activity of inducible iNOS. They demonstrated that LPS administration markedly increased iNOS expression and NO production in liver and lung tissues, contributing to systemic inflammation. However, melatonin significantly counteracted these effects by reducing iNOS mRNA and protein levels, as well as NO production (Escames et al., 2003). A study showed that in LPS-induced septic skeletal muscle, melatonin preserves mitochondrial function by maintaining the activity of respiratory chain complexes I and IV, restoring ATP production, and stabilizing mitochondrial membrane potential. It reduces mitochondrial oxidative stress by scavenging ROS and upregulating antioxidant enzymes such as SOD2 and GPx1. Melatonin also prevents the opening of the mitochondrial permeability transition pore (mPTP) and activates AMPK, promoting cellular energy balance and mitochondrial biogenesis (Ozkok et al., 2016). Wu et al. investigated the therapeutic potential of melatonin in a rat model of peritonitis-induced septic shock and multiple organ dysfunction syndrome (Wu et al., 2008). They demonstrated that melatonin (3 mg/kg, administered intravenously at 3, 6, and 12 h post-cecal ligation and puncture) significantly improved survival, stabilized blood pressure, restored vascular responsiveness to norepinephrine, and reduced organ injury markers. Melatonin treatment suppressed inflammatory mediators such as interleukin-1β and NO, decreased superoxide production in the aorta, and mitigated polymorphonuclear neutrophil (PMN) infiltration in the lungs and liver (Wu et al., 2008). In addition, a clinical study by El-Gendy et al. reported significant reductions in inflammatory markers and oxidative stress among neonates with sepsis who received melatonin as adjunctive therapy, along with a decrease in mortality risk (El-Gendy et al., 2018). These findings were reinforced by a systematic review and meta-analysis by Henderson et al., which consolidated data from multiple neonatal studies and concluded that melatonin supplementation was associated with reduced oxidative damage and improved clinical outcomes (Henderson et al., 2018).

#### Omega-3 fatty acid supplementation

Omega-3 polyunsaturated fatty acids (PUFAs), particularly eicosapentaenoic acid (EPA) and docosahexaenoic acid (DHA) from fish oil, have demonstrated therapeutic relevance in modulating critical inflammatory and immune pathways implicated in septic progression (Shahidi and Ambigaipalan, 2018). By altering lipid mediator profiles, dampening cytokine cascades, and enhancing cellular resilience, omega-3 supplementation has shown potential to attenuate systemic inflammation and improve clinical outcomes in septic patients (Okut et al., 2025). Tseng et al. conducted a network meta-analysis demonstrating that high-dose omega-3 PUFAs were associated with improved clinical outcomes in septic patients, including reduced mortality and organ dysfunction, with favorable safety and acceptability profiles (Tseng et al., 2024). Similarly, Wang et al. reported in their meta-analysis that fish oil-enriched nutrition reduced ICU length of stay and improved overall prognosis when compared to standard nutrition in adult sepsis patients (Wang H. et al., 2022). Furthermore, a meta-analysis of 20 randomized controlled trials found that omega-3 fatty acid supplementation was associated with reduced mortality, shorter mechanical ventilation duration, and decreased ICU stay in patients with sepsis, with the greatest benefit observed in those with gastrointestinal dysfunction (Wang et al., 2020). Chen et al. examined the effect of omega-3 fatty acids on mortality in adults with sepsis and sepsis-induced ARDS. While some benefit was observed with enteral use, the overall impact on mortality was not statistically significant. Results suggest potential but remain inconclusive (Chen et al., 2018). Nevertheless, Omega-3 PUFAs exert their effects through modulation of the eicosanoid pathway, replacing arachidonic acid in cell membranes and subsequently reducing synthesis of pro-inflammatory mediators such as leukotriene B4 and prostaglandin E2 (Calviello et al., 2013; Lu et al., 2017). In terms of administration, both enteral and parenteral routes have demonstrated efficacy. A study by Chen et al. showed that enteral omega-3 fish oil reduced mortality in patients with severe sepsis and acute gastrointestinal injury, underscoring the gut’s role as a critical immunological interface in critical illness (Chen et al., 2017). Another trial using intravenous omega-3 administration reported beneficial modulation of plasma phospholipid profiles and inflammatory markers during early sepsis (Hosny et al., 2013). Recent experimental work also supports these findings. Velasque et al. demonstrated that omega-3-rich fish oil supplementation protected liver tissue from oxidative stress and injury in a rodent sepsis model, suggesting organ-specific cytoprotective effects that extend beyond systemic immunomodulation (Velasque et al., 2023).

#### Selenium

Selenium is an essential trace element critical for the function of various antioxidant enzymes, including glutathione peroxidases and thioredoxin reductases, and is investigated as a therapeutic adjunct in sepsis (Table 4) (Bai et al., 2024). In critically ill patients, plasma selenium levels are frequently depleted, correlating with heightened oxidative stress, impaired immune responses, and increased mortality risk. Restoring selenium homeostasis in this context has emerged as a targeted strategy to counteract sepsis-associated oxidative injury and immune dysregulation (Landucci et al., 2014). Prabhu et al. found that selenium deficiency in RAW 264.7 macrophages increases oxidative stress and enhances iNOS expression and NO production through heightened NF-κB activation, suggesting selenium’s key role in regulating inflammatory responses (Prabhu et al., 2002). A trial tested high-dose sodium selenite in 249 ICU patients with severe SIRS, sepsis, or septic shock. While mortality reduction in the full cohort was nonsignificant, the per-protocol group showed a significant 28-day survival benefit (42.4% vs. 56.7%). Benefits were strongest in patients with septic shock, high APACHE III scores, and lower multiple organ failure. Selenium supplementation increased blood selenium and glutathione peroxidase-3 activity without side effects, supporting it as a safe, low-cost adjuvant therapy in severe sepsis (Angstwurm et al., 2007). Another clinical trial enrolled 150 ICU patients with SIRS or sepsis to evaluate high-dose selenium (1,000 µg on day 1, then 500 µg/day for 14 days) *versus* standard supplementation. High-dose selenium significantly increased plasma selenium and glutathione peroxidase activity and was associated with reduced inflammatory markers and improved nutritional indices (prealbumin, cholesterol) (Valenta et al., 2011). More recent findings by Li et al. confirmed selenium’s potential benefits in reducing ICU and hospital stay durations, vasopressor dependency, and the incidence of nosocomial infections, reinforcing its role in improving clinical trajectories (Li et al., 2019). Kwon et al. demonstrated that selenium combined with niacin significantly reduced sepsis-induced lung injury *via* activation of the Nrf2 signaling pathway (Kwon et al., 2016). Kočan et al. investigated the effects of adjunctive selenium therapy in 65 septic patients stratified by oxygenation status (PaO_2_/FiO_2_ ≥200 or <200). Participants received continuous intravenous sodium selenite (750 µg/day for 6 days) or placebo. Selenium administration significantly enhanced glutathione peroxidase activity and improved oxygenation in patients with severe respiratory failure (PaO_2_/FiO_2_ <200), indicating potential pulmonary and antioxidant benefits, though without a significant impact on overall mortality (Kočan et al., 2014). Chelkeba et al. further supports selenium’s therapeutic value, showing improved outcomes in mechanically ventilated septic patients when selenium was added to standard care protocols (Chelkeba et al., 2015). Furthermore, Barchielli et al. showed that selenium, *via* key selenoproteins (GPx, TrxR, SelP), is essential for redox balance, thyroid function, immunity, fertility, and disease prevention. Adequate intake protects against cancer, cardiovascular, neurodegenerative, and inflammatory diseases, while deficiency worsens outcomes. However, excess selenium can be toxic, though its pro-oxidant properties may be exploited in cancer therapy (Barchielli et al., 2022).

#### Carnosine

Carnosine (β-alanyl-L-histidine), a naturally occurring dipeptide abundant in skeletal muscle and the brain, has gained interest as a multifunctional cytoprotective agent due to its antioxidant, anti-inflammatory, and metal-chelating properties (Caruso, 2022). In sepsis, carnosine has shown promise in several preclinical studies as a potential therapeutic intervention (Table 4). Experimental studies have demonstrated that carnosine alleviates sepsis-induced ALI by reducing oxidative stress and modulating inflammatory responses. In a rat model, carnosine significantly attenuated pulmonary damage, downregulated NF-κB activation, and improved overall survival rates, highlighting its protective role in septic lung pathology (Tanaka et al., 2017; Sun et al., 2017). Beyond pulmonary effects, carnosine demonstrates renoprotective actions in sepsis. In models of sepsis-induced acute kidney injury, carnosine administration mitigated tubular damage, suppressed pro-inflammatory cytokines, and improved renal function markers (Sahin and Burukoglu Donmez, 2018; Wang et al., 2021). Mechanistically, carnosine exerts its effects through antioxidant and carbonyl scavenging activity, thereby reducing lipid peroxidation and protein carbonylation. It also modulates inflammatory signaling pathways, helping restore immune balance during septic progression (Sahin et al., 2013; Chmielewska et al., 2020). Interestingly, carnosine metabolism may also generate anserine, a derivative that reduces capillary leakage and mortality in experimental sepsis, further supporting its translational potential (Table 5) (Schmoch et al., 2025).

**TABLE 5:** Overview of key antioxidant systems and their mechanistic roles in the pathogenesis and modulation of sepsis.

Antioxidant agent	Subcellular/Physiological localization	Mechanism of action	References
Superoxide dismutase (SOD)	Cytosol, mitochondria, peroxisomes, chloroplasts	Converts superoxide radicals (O_2_^−^) to hydrogen peroxide (H_2_O_2_) and molecular oxygen (O_2_), thereby mitigating mitochondrial and cytosolic oxidative stress.	Bao et al. (2022), Ismy et al. (2021)
N-Acetylcysteine (NAC)	Administered orally or topically	Precursor for glutathione biosynthesis; acts as a direct scavenger of free radicals and supports methylation metabolism.	Chertoff (2018), de Mello et al. (2011)
Catalase	Peroxisomes, mitochondria	Catalyzes decomposition of hydrogen peroxide into water and oxygen, reducing peroxidative damage.	Ayar et al. (2017), Shimozaw et al. (2000)
Glutathione	Cytosol, mitochondria, chloroplasts	Detoxifies hydrogen peroxide and lipid hydroperoxides *via* reduced glutathione, maintaining redox equilibrium.	Biolo et al. (2007), Li et al. (2018)
Vitamin A (retinoids)	Chloroplasts	Inhibits NF-κB activation, reduces ROS *via* β-carotene, and promotes Nrf2-dependent antioxidant gene transcription.	Cherukuri et al. (2019), Zha et al. (2019)
Vitamin C	Cytosol, mitochondria, peroxisomes, chloroplasts	Regenerates oxidized Vitamin E, scavenges ROS/RNS, inhibits pro-inflammatory mediators and nitric oxide synthase (NOS) isoforms.	Marik et al. (2017), Ammar et al. (2021)
Vitamin E	Plasma membrane	Interrupts lipid peroxidation chains, enhances nitric oxide bioavailability, and protects endothelial function.	Dang et al. (2021), Thompson et al. (2022)
Melatonin	Pineal gland	Enhances electron transport chain efficiency, reducing ROS formation; exhibits both direct and indirect antioxidative effects.	Deng et al. (2024), Acuña-Castroviejo et al. (2017)
Omega-3 fatty acids	Incorporated into cellular membranes (especially immune cells)	Modulates oxidative stress and inflammation by altering membrane fluidity, reducing ROS production, and generating specialized pro-resolving lipid mediators (e.g., resolvins).	Chen et al. (2023), Chen et al. (2022), Erdem et al. (2023)
Selenium	Incorporated into selenoproteins (e.g., GPx, thioredoxin reductase)	Enhances antioxidant defenses by supporting selenoenzyme activity; reduces lipid peroxidation and inflammatory signaling during septic progression.	Belsky et al. (2018), Chen and Thiemermann (2016)
Carnosine	High concentrations in muscle, brain, and heart tissues	Acts as a metal chelator, pH buffer, and scavenger of reactive aldehydes and ROS; modulates nitric oxide metabolism and cytokine production.	Wang et al. (2021), Sahin et al. (2013)

## Therapeutic limitations and risks associated with antioxidant treatment

The therapeutic use of antioxidants in sepsis has gained significant attention due to the well-established role of oxidative stress in the pathogenesis of this condition. Despite promising mechanistic insights and preclinical data, clinical translation has faced substantial limitations and risks that restrict widespread implementation. Previous antioxidant trials in critical illness, especially sepsis, have largely failed due to challenges in pharmacokinetics, patient selection, and trial design (Table 6). Antioxidants often exhibit poor bioavailability, rapid clearance, and inadequate tissue distribution in critically ill patients, making it difficult to achieve therapeutic concentrations at the right time. Additionally, most trials enrolled unstratified, heterogeneous patient populations without using oxidative stress biomarkers to identify individuals most likely to benefit. This lack of precision diluted any potential therapeutic signal. Comorbidities such as diabetes or liver dysfunction further distorted redox balance and response. While antioxidants such as vitamins C and E, melatonin, N-acetylcysteine, and mitochondrial-targeted compounds have shown beneficial effects in experimental models, their clinical efficacy remains inconsistent or inconclusive in humans. Many studies highlight the absence of statistically significant outcomes or improvement in mortality among septic patients receiving antioxidants, which undermines their routine clinical use (Prauchner, 2017; Galley, 2010). For example, HDIVC (50 mg/kg every 6 h for 96 h) failed to improve outcomes in vasopressor-treated septic ICU patients, instead increasing death or persistent organ dysfunction without secondary benefits (Lamontagne et al., 2022). Moreover, transcriptomic analysis from the LOVIT trial revealed that vitamin C lowered mortality in adaptive but worsened outcomes in inflammopathic sepsis, suggesting immune profiling may guide personalized therapy (Rynne et al., 2025). In addition, Manapurath et al. conducted a systematic review and meta-analysis of four trials involving 800 very low birth weight or very preterm infants to evaluate the effects of low-dose enteral vitamin A supplementation (≤10,000 IU/day). While supplementation significantly increased serum retinol levels, it had no significant effect on sepsis incidence. Despite the biological plausibility of vitamin A in supporting immune function, the findings do not currently support its use for sepsis prevention in this population (Manapurath et al., 2022). Another critical limitation lies in the complexity of dosing and timing. The optimal therapeutic window for antioxidant administration in sepsis remains poorly defined. Delayed treatment may fail to mitigate the oxidative cascade, while early intervention could interfere with beneficial immune responses that rely on ROS for pathogen clearance (Forman and Zhang, 2021). Additionally, inter-patient variability in oxidative stress levels complicates the establishment of standardized antioxidant protocols (Berger and Chioléro, 2007). From a risk perspective, antioxidants may exert unintended immunosuppressive effects. By scavenging ROS, antioxidants can potentially impair neutrophil and macrophage function, thereby weakening the host defence mechanisms critical in septic patients (Galley, 2010). Moreover, selenium and other trace element supplements, though biologically active, carry a narrow therapeutic window, and overdosing could exacerbate organ dysfunction or induce toxicity (Üstündağ, 2023). A phase II trial evaluated high-dose selenium (4,000 μg day 1, then 1,000 µg/day for 9 days) in 60 septic shock patients. Continuous infusion was well tolerated but showed no improvement in mortality or organ support duration, suggesting bolus dosing may be more effective (Forceville, 2007). A meta-analysis by Alhazzani et al. concluded that selenium supplementation, particularly in the form of intravenous sodium selenite, may reduce mortality in septic patients, although the evidence was heterogeneous and modest in effect size (Alhazzani et al., 2013). Finally, combination antioxidant therapies, while conceptually promising, present challenges in pharmacokinetics, potential interactions, and evaluation of synergistic effects. Trials employing multitherapy antioxidant regimens have encountered difficulties in isolating specific contributions of individual components and ensuring safe, effective dosage ratios (Abelli et al., 2022).

**TABLE 6:** Key limitations and risks of antioxidant therapies in sepsis.

Antioxidant agent	Key limitations and risks	Underlying mechanism	References
SOD Mimetics	Pro-oxidant effects; narrow therapeutic window.	Overwhelms H_2_O_2_ detoxification systems (CAT/GPx).	Murphy et al. (2008), Batinić-Haberle et al. (2010)
N-Acetylcysteine (NAC)	Pro-oxidant activity; anaphylactoid reactions.	Reduces Fe^3+^ to Fe^2+^, potentiating Fenton chemistry.	Rushworth and Megson (2014), Raghu et al. (2021)
High-Dose Vitamin C	Oxalate nephropathy; assay interference.	Metabolic conversion to oxalate crystals in renal tubules.	Shen et al. (2023), Xu et al. (2023)
Vitamin E	Increased all-cause mortality; anticoagulant effect.	Inhibition of platelet aggregation and vitamin K-dependent carboxylase.	Miller et al. (2005), Schürks et al. (2010)
Selenium	Selenosis; narrow therapeutic index.	Non-specific incorporation into proteins at high doses.	Vinceti et al. (2018), Roman et al. (2014)
Omega-3 Fatty Acids	Immunosuppression; enhanced lipid peroxidation.	Modulation of immune signaling; peroxidation of PUFAs.	Fritsche (2015), Gutiérrez et al. (2019)
Melatonin	Sedation; limited robust RCT data.	Agonism of MT_1_/MT_2_ receptors in the CNS.	Andersen et al. (2016), Seabra et al. (2000)
Vitamin A	Hypervitaminosis A; hepatotoxicity; teratogenicity.	Storage in hepatic stellate cells leading to cytotoxicity.	Penniston and Tanumihardjo (2006), Myhre et al. (2003)
Carnosine	Rapid hydrolysis; low bioavailability.	Degradation by serum carnosinase (CN1).	Boldyrev et al. (2013), Gardner et al. (1991)

## Future directions

Although current antioxidant strategies in sepsis have yielded mixed clinical results, evolving insights into the disease’s redox biology are shaping a new generation of targeted and personalized interventions. Several future directions are emerging to overcome past limitations and optimize the therapeutic potential of antioxidants in sepsis. A critical area of focus is the targeted delivery of antioxidants to mitochondria, the primary source of ROS in sepsis-induced organ dysfunction. Mitochondria-targeted compounds, such as MitoQ and SkQ derivatives, are being investigated for their ability to localize antioxidant effects precisely where oxidative damage is most profound (Víctor et al., 2009). In parallel, natural antioxidants and phytochemicals (e.g., polyphenols, flavonoids) are gaining traction due to their multifaceted properties, including anti-inflammatory, antimicrobial, and antioxidant activities. These agents may provide a safer profile compared to synthetic antioxidants, especially when used in synergistic formulations (Üstündağ, 2023; Shrivastava et al., 2023). Biomarker-guided therapy is another promising avenue. Oxidative stress levels vary among patients, and identifying reliable biomarkers (e.g., isoprostanes, nitrotyrosine, mitochondrial DNA) could allow stratification of patients who are most likely to benefit from antioxidant treatment. This precision medicine approach may also guide dosage and timing, two factors previously identified as critical to therapeutic success (Santacroce et al., 2024). Furthermore, emerging technologies such as nanomedicine and nano-antioxidants offer new platforms for antioxidant delivery. Nanocarriers can encapsulate antioxidants, enhance bioavailability, and direct compounds to specific tissues or cells involved in sepsis pathology. Some nanomaterials even possess inherent redox-modulating properties, blurring the lines between vehicle and therapy (Lin et al., 2024). Guo et al. demonstrated that nano-parthenolide improved survival and intestinal barrier function in septic rats by reducing ROS and apoptosis through 5-HTR2A regulation, outperforming conventional formulations (Guo et al., 2023). Li et al. designed multifunctional nanoparticles capable of simultaneously scavenging LPS, ROS, and cell-free DNA, effectively suppressing cytokine storms and reducing mortality in animal sepsis models (Li et al., 2023b). Extending beyond systemic inflammation, Qu et al. developed a biomimetic nanomodulator targeting sepsis-associated encephalopathy, which crossed the blood-brain barrier, alleviated oxidative stress, polarized macrophages toward anti-inflammatory phenotypes, and improved cognition and survival (Qu et al., 2024). Complementing antioxidant-based strategies, Tang et al. introduced mRNA-lipid nanoparticles that reprogrammed macrophages *in situ* into CAR-MΦs, restoring immune clearance against multidrug-resistant bacteria in septic mice (Tang et al., 2024). Finally, the integration of artificial intelligence and real-time monitoring presents a transformative opportunity; developing machine learning algorithms that dynamically adjust antioxidant infusion based on continuous clinical data could enable adaptive, personalized dosing, while federated learning models could predict patients at high risk for oxidative organ failure, facilitating pre-emptive intervention. Alam et al. proposed FedSepsis, a federated deep-learning framework using electronic health records and IoMT devices for early sepsis detection, underscoring the potential of integrating predictive analytics with antioxidant-based therapeutics (Alam and Rahmani, 2023). Collectively, these directions point toward multifunctional nanotherapies, precision redox modulation, and AI-driven early detection systems as transformative approaches for harnessing antioxidants in sepsis management.

## Conclusion

The intricate interplay among oxidative stress, mitochondrial dysfunction, and immune dysregulation constitutes a central axis in the pathogenesis of sepsis. Although antioxidant-based therapies are biologically compelling, their clinical application remains challenged by variability in efficacy, timing of administration, and patient-specific factors. Among emerging strategies, mitochondria-targeted antioxidants such as melatonin and MitoQ, along with adjunctive agents like N-acetylcysteine and vitamin C, offer promising therapeutic avenues. Moreover, biomarker-guided treatment and nanomedicine-enabled delivery systems provide a foundation for precision medicine approaches that could enhance both safety and effectiveness. Moving forward, integrated efforts to establish reliable biomarkers, refine dosing protocols, and align interventions with distinct immunometabolic endotypes are essential. Such a mechanistically informed and patient-tailored strategy may ultimately shift sepsis management from generalized supportive care toward targeted, disease-modifying therapy.
